# Dengue — Quo tu et quo vadis?

**DOI:** 10.3390/v3091562

**Published:** 2011-09-01

**Authors:** Rubing Chen, Nikos Vasilakis

**Affiliations:** 1 Department of Pathology, University of Texas Medical Branch, 301 University Blvd, Galveston, TX 77555, USA; E-Mail: ruchen@utmb.edu; 2 Center for Biodefense and Emerging Infectious Diseases, University of Texas Medical Branch, 301 University Blvd, Galveston, TX 77555, USA; 3 Institute for Human Infection and Immunity, University of Texas Medical Branch, 301 University Blvd, Galveston, TX 77555, USA; 4 Center for Tropical Diseases, University of Texas Medical Branch, 301 University Blvd, Galveston, TX 77555, USA

**Keywords:** dengue virus (DENV), arbovirus, mosquito, evolution, phylogenetics

## Abstract

Dengue viruses (DENV) are by far the most important arboviral pathogens in the tropics around the world, putting at risk of infection nearly a third of the global human population. DENV are members of the genus *Flavivirus* in the Family *Flaviviridae* and comprise four antigenically distinct serotypes (DENV-1-4). Although they share almost identical epidemiological features, they are genetically distinct. Phylogenetic analyses have revealed valuable insights into the origins, epidemiology and the forces that shape DENV evolution in nature. In this review, we examine the current status of DENV evolution, including but not limited to rates of evolution, selection pressures, population sizes and evolutionary constraints, and we discuss how these factors influence transmission, pathogenesis and emergence.

## 1. Introduction

**Figure 1.**
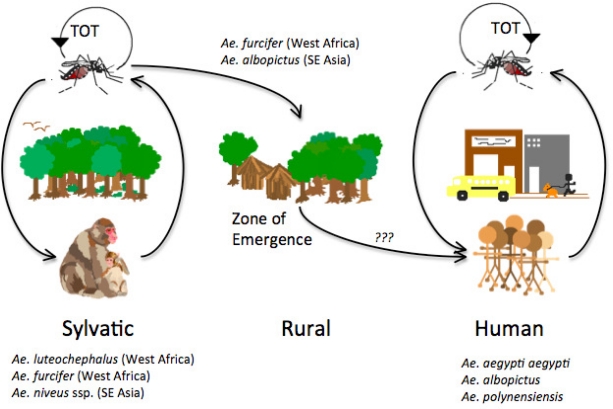
The Transmission Cycles of Dengue Virus (DENV). The transmission cycles of DENV, depicting the sylvatic origins and the ‘zone of emergence’ where sylvatic cycles contact human populations in rural areas in West Africa and Southeast Asia. Image courtesy of Shannan Rossi, Department of Pathology, UTMB.

Dengue viruses (DENV) are arthropod-borne viruses (arboviruses) in the genus *Flavivirus* (family *Flaviviridae*) with positive polarity, single-stranded RNA. They utilize *Aedes* (Stegomyia) spp., primarily *Ae. aegypti* and *Ae. albopictus*, as vectors for domestic and peridomestic transmission, and arboreal *Aedes* mosquitoes as vectors for enzootic transmission (Figure 1). All DENV group into four genetically related but antigenically distinct serotypes (DENV-1, -2, -3 and -4) within the dengue (DEN) antigenic complex [1]. They are extremely restricted in their natural vertebrate host range, which includes only primates [2,3], although some reports suggest for a putative, unconfirmed, extended vertebrate host range [4–6]. After World War II there was an explosive increase in the geographic distribution of all four DENV serotypes fueled by uncontrolled urbanization, rapid population movement facilitated by jet travel, inadequate water, sewer and waste management, as well as unsustainable vector control programs [7]. At present, all four DENV serotypes can be found in nearly all urban and peri-urban environments throughout the tropics and subtropics where *Aedes* (Stegomyia) *aegypti aegypti* and *Ae*. (Stegomyia) *albopictus* are present. Thus the global distribution of these two major vectors puts nearly a third of the global human population at risk of infection [8]. Currently DEN has become endemic in more than 100 countries and the disease is spreading to new areas where outbreaks take place in immunologically naïve populations [9,10].

By current estimates, the impact of DENV infections on human health is enormous; DENV are responsible for *ca*. 100 million infections per year presenting in a spectrum of clinical manifestations. While the great majority of infections are accompanied by little no or subclinical signs and symptoms, symptomatic infections commonly manifest as a self-limited flu-like disease [dengue fever (DF)] characterized by a sudden onset of fever, arthralgia, myalgia, retro-orbital headaches, maculopapular rash, and leucopenia. Approximately 1–2% of human infections present clinically as the most severe form of the disease [dengue hemorrhagic fever/dengue shock syndrome (DHF/DSS)], characterized by vascular leakage and/or hemorrhage, leading to *ca.* 500,000 annual hospitalizations with a case fatality rate of about 5% [11]. Although severe DEN disease is historically associated with pediatric populations in hyperendemic regions (areas in which circulation of all 4 DENV serotypes takes place) [12–14], recent trends from Southeast Asia and Latin America suggest that adults may also be at risk [15–23]. Risk factors for the development of severe DEN disease include prior infection with a heterotypic serotype [24–26], the strain of the infecting virus [27–29], age and gender [30,31], nutritional status [32,33] and the genetic background of the patient [34–36]. Because of underreporting of disease and under-utilization of health services especially in resource-poor countries, the true health and economic burden due to DENV infections is difficult to estimate [37]. Nonetheless, it is estimated that in the endemic areas of Asia and the Americas, the burden of DEN is in the range 150–1,300 disability adjusted life years (DALY) per million, depending on the spatiotemporal attributes of the epidemics [8,38–42] (reviewed in [43]), and the costs associated with a DENV infection significantly exceed the average monthly income of the patient [38,42,44–52].

In this review, we examine the current status of DENV evolution, including but not limited to rates of evolution, selection pressures, population sizes and evolutionary constraints, and discuss how these factors influence transmission, pathogenesis and emergence.

## 2. History, Origin and Emergence of DENV

DENV undoubtedly have a long history of infecting humans. The earliest known references date back to the Chin, Tang and Northern Sung Dynasties [Common Era (CE) 265–420, 610 and 992 AD respectively] describing a disease referred to as ‘water poison,’ clearly for its association with water-associated insects [7,53]. Since *Ae. aegypti aegypti*, the primary vector of DENV transmission has been introduced in Asia through the sailing ship trade a few hundred years ago, the likely vector of transmission at that time would have been *Ae. albopictus*. The next available reports first appeared in the 17th Century and for the next two centuries describe a disease with widespread geographic distribution fueled by the sailing ship and slave trade, which reached pandemic proportions by the late 18th Century [54–57] (reviewed in [7,58]). Although the clinical manifestations are consistent with a DEN-like disease, including fever, rash, arthralgia, myalgia and hemorrhagic manifestations, it is possible that the described illness was due to other aetiologic agents [for example chikungunya virus (CHIKV)] that produce illness often indistinguishable from DEN [54,59,60].

**Figure 2.**
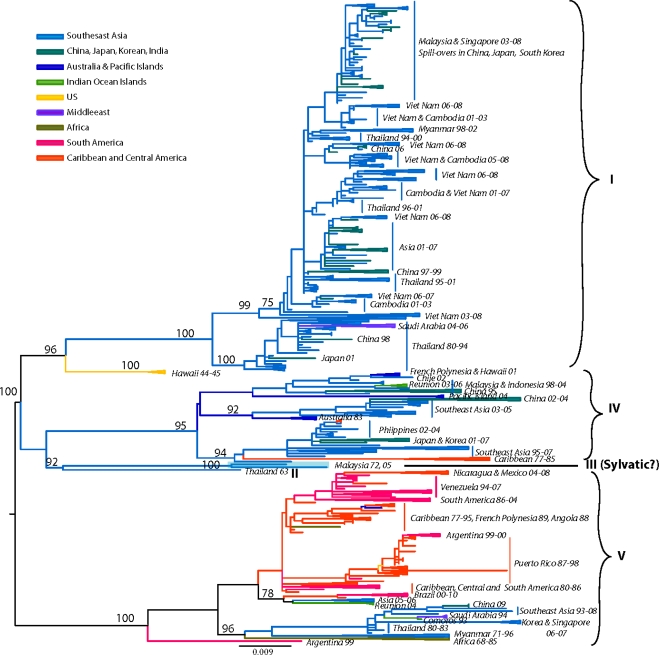
Dengue Virus Type 1 Phylogeny. Maximum Likelihood Tree (MLT) of DENV-1 based on complete E gene nucleotide sequences of all available naturally occurring strains (n = 1812) from GenBank. MLT was reconstructed using PAUP* version 4b10-MacOsX package [161], employed the NNI branch swapping method, and based on the best-fit nucleotide substitution model estimated by MODELTEST [162]. Bootstrap values (NJ method with 1000 replications) above 70 were labeled along the branch of major clades. The tree is mid-point rooted. Color lines represent specific geographic regions as specified within the figure.

**Figure 3.**
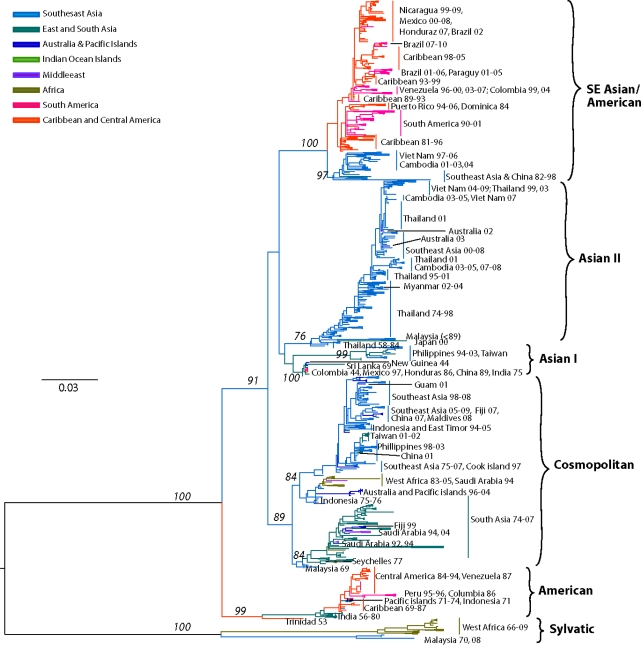
Dengue Virus Type 2 Phylogeny. Maximum Likelihood Tree (MLT) of DENV-2 based on complete E gene nucleotide sequences of all available naturally occurring strains (n = 1827) from GenBank. MLT was reconstructed using PAUP* version 4b10-MacOsX package [161], employed the NNI branch swapping method, and based on the best-fit nucleotide substitution model estimated by MODELTEST [162]. Bootstrap values (NJ method with 1000 replications) above 70 were labeled along the branch of major clades. The tree is mid-point rooted. Color lines represent specific geographic regions as specified within figure.

**Figure 4.**
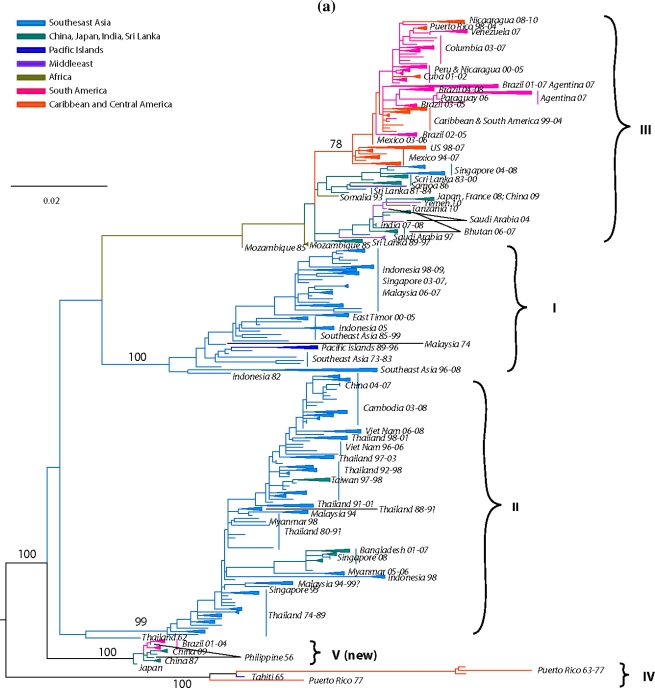

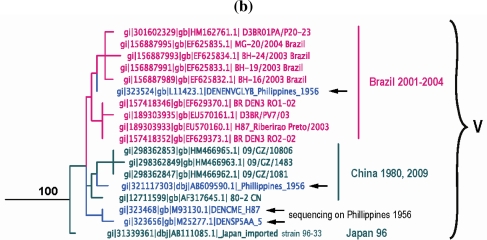
(**a**) Dengue Virus Type 3 Phylogeny. Maximum Likelihood Tree (MLT) of DENV-3 based on complete E gene nucleotide sequences of all available naturally occurring strains (n = 1208) from GenBank. MLT was reconstructed using PAUP* version 4b10-MacOsX package [161], employing the NNI branch swapping method, and based on the best-fit nucleotide substitution model estimated by MODELTEST [162]. Bootstrap values (NJ method with 1000 replications) above 70 were labeled along the branch of major clades. The tree is mid-point rooted. Color lines represent specific geographic regions as specified within figure. (**b**) Genotype V lineage discrepancies.

**Figure 5.**
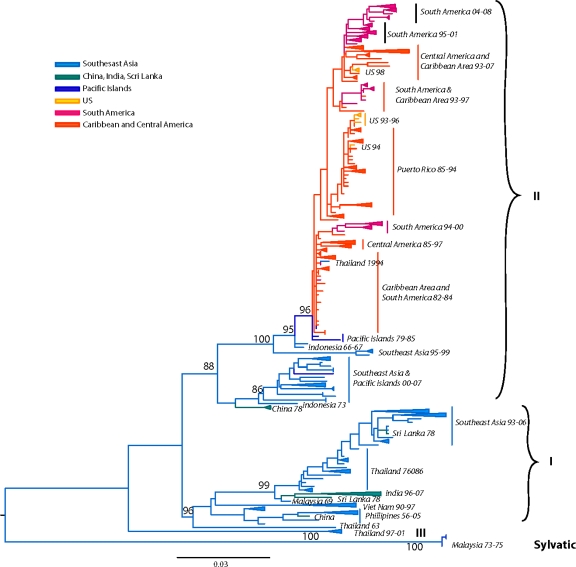
Dengue Virus Type 4 Phylogeny. Maximum Likelihood Tree (MLT) of DENV-4 based on complete E gene nucleotide sequences of all available naturally occurring strains (n = 418) from GenBank. MLT was reconstructed using PAUP* version 4b10-MacOsX package [161], employed the NNI branch swapping method, and based on the best-fit nucleotide substitution model estimated by MODELTEST [162]. Bootstrap values (NJ method with 1000 replications) above 70 were labeled along the branch of major clades. The tree is mid-point rooted. Color lines represent specific geographic regions as specified within figure.

Many aspects of the origin and evolution of DENV remain unclear to this day. It has been hypothesized that the human DENV that circulate widely today in urban and peri-urban centers throughout the tropics and neotropics evolved from sylvatic progenitors [7,61]. Phylogenetic analyses of available DENV gene sequences have shed some light on DENV evolutionary history [58,62–70] (Figures 2–5). These analyses indicate that all current distinct DENV serotypes evolved independently and repeatedly in a series of divergence events that occurred after the establishment of large enough urban populations to support the human transmission cycle [63,64,67,71,72]. This emergence process was facilitated through vector switching, from arboreal *Aedes* mosquitoes to peridomestic (*Ae. albopictus*) and domestic (*Ae. aegypti aegypti*) mosquitoes and switching of reservoir hosts from non-human primates to humans (Figure 1). Furthermore, the emergence of the extant, distinct human DENV strains from the sylvatic transmission cycle was most likely facilitated by the allopatric and perhaps ecological partitioning of ancestral sylvatic DENV strains in different species of non-human primate populations. The onset of transoceanic exploration in the 15th Century and the subsequent establishment of trading routes and waves of human immigration provided the means for the dispersal of the four DENV sylvatic serotypes and the elimination of their allopatric distributions. By then the four serotypes had diverged antigenically to support the limited heterotypic cross-protection against challenge exhibited by current strains of DENV [73,74]. This reality evidently released the four serotypes from direct competition for susceptible hosts, thus allowing for their sympatry due to incomplete cross-protection, which presumably reduce competition for a common host population. Therefore, the currently observed coexistence of multiple serotypes with their extensive genetic diversity may have been and may still be selected by immune enhancement (enhancement of virus replication following heterologous infection). In such settings, each virus gains replication and transmission efficiency in the human and/or non-human primate host due to limited cross-reactive immunity from previous, heterologous DENV infections [75–78] (SEE ‘Cross-Immunity as a Driving Force for DENV Evolution’ AND ‘Antibody-Dependent Enhancement (ADE) and Its Effect on DENV Evolution’ sections below). Indeed, several lines of evidence support the role of immune enhancement as a factor in the observed oscillations of DEN incidence [79,80]. Selection for higher virus replication facilitated by immune enhancement also may influence higher transmissibility by the mosquito vector. Since *Ae. aegypti aegypti* is only moderately susceptible to DENV infection, selection of strains of higher fitness and thus higher viremia in the vertebrate host may have enhanced its capacity to spread these in new territories and displace endemic strains of lower fitness and pathogenic potential [77,81–83]. Thus, even if a vector is highly susceptible and the infection threshold is well below the typical viremia peak titer, higher viremia is usually accompanied by longer viremia such that the amount of time an individual is infectious for a vector is extended.

The geographic origin of DENV has been subject to speculation and debate for decades. Based on the available data it is impossible to conclusively identify Asia *versus* Africa as the origin of DENV. Some have proposed the ultimate origins of DENV in Africa, based on the circulation there of several closely related mosquito-borne flaviviruses [84] and/or the African origin the principal DENV vector, *Ae. aegypti aegypti* [85]. The argument for the African origin of DENV parallels that of yellow fever virus (YFV), which is also vectored in its human cycle by the same mosquito species, and was introduced into the Americas during the slave trade *ca.* 300–400 years ago, where it established sylvatic cycles that persist today [86] and which repeatedly seeded spillover epidemics into the human transmission cycle [87,88]. Gordon Smith suggested the African origin of DENV based on the African origin of *Ae. aegypti aegypti* which was supported by the following lines of evidence: (i) abundance in Africa of the closely related *Aedes* (Stegomyia) spp.; (ii) the absence of related *Aedes* (Stegomyia) spp. in the Americas, and (iii) the Africa-only existence of sylvatic, ancestral *Ae. aegypti formosus* [89,90]. However, as discussed below (see ‘Transmission Cycles’ section) sylvatic DENV circulating in Africa do not utilize *Ae. aegypti formosus* as a vector, and recent evidence indicates this subspecies is refractory to DENV infection [91–93]. Wang *et al.* [63] proposed an ‘out of Asia’ origin of DENV, based on the following ecologic and phylogenetic evidence. First, all 4 sylvatic DENV serotypes circulate in Southeast Asia [although there is only serologic evidence of sylvatic DENV-3 circulation and the phylogeny of sylvatic DENV-1 is uncertain (see Sections 4.1 and 4.3)] [2,58,63,69,94], whereas only sylvatic DENV-2 transmission has been documented in Africa [95–97]. Second, extensive phylogenetic analyses demonstrate the deep phylogenetic positions of the Asian sylvatic strains [58,63,65,70,94,98]. Lastly, *Ae. albopictus*, a peridomestic mosquito of Asian origin but only a secondary vector for human DENV transmission [99], has been shown in experimental studies to be more susceptible to DENV virus infection than *Ae. aegypti aegypti* [100]. Furthermore, early serologic studies (neutralizing antibodies) in Southeast Asia in human populations inhabiting a variety of ecologic habitats devoid of *Ae. aegypti aegypti* [85] suggest that *Ae. albopictus* was the original vector for human DENV and that the virus exploited the anthropophagic nature and the extended geographic distribution of the domestic *Ae. aegypti aegypti* to facilitate the sustained, yet explosive, transmission among humans [7,101–103]. Regardless of the arguments presented above, it is clear that a full understanding of the origins and evolutionary history of DENV will require a far larger sample of sylvatic viruses than is currently available. Furthermore, our understanding of DENV origins may be enhanced by extensive metagenomic surveys of flavivirus diversity, since we have undoubtedly only sampled a small fraction of the total diversity of flaviviruses [68–70,84,94,104].

## 3. Transmission Cycles

The four DENV serotypes are maintained in two distinct transmission cycles: (a) sylvatic and (b) human (Figure 1).

The sylvatic cycle is ecologically and evolutionarily distinct from the human transmission cycle. It takes place in the sylvan environments of Southeast Asia and West Africa in well-documented foci in peninsular Malaysia [2] and eastern Senegal [95,105], respectively. Recent phylogenetic analyses have extended the spatiotemporal range in which sylvatic DENV are known to circulate in West Africa [97]. In this cycle, transmission is mediated by arboreal canopy-dwelling *Aedes* spp. and non-human primates appear to be the only amplification and reservoir hosts. In Africa, the principal vectors include *Ae.* (Stegomyia) *luteocephalus*, *Ae.* (Diceromyia) *furcifer*, and *Ae.* (Diceromyia) *taylori* (Figure 1) [95,96,105–107]. Although *Ae. furcifer* are primarily canopy-dwelling enzootic mosquitoes, they are known to descend to ground level to feed on humans [105]. Surprisingly, the arboreal, ancestral form, *Ae. aegypti formosus*, of the major domestic vector for DENV transmission (see below), is refractory to sylvatic DENV infection [91,92,105]. The primate reservoir hosts in Africa include the Patas monkey (*Erythrocebus patas*), African green monkey (*Chlorocebus sabaeus*), Guinea baboon (*Papio papio*) [3,108] and possibly related species such as *Papio anubis*, *Papio ursinus and Papio cynocephalus* [94]. In Asia, the principle vectors include the primatophilic canopy-dwelling mosquitoes of the *Ae.* (Finlaya) *niveus s.l*. complex, a group that includes *Ae. pseudoniveus*, *Ae. subniveus*, *Ae. vanus*, *Ae. albolateralis*, *Ae. niveoides* and *Ae. novoniveus* [2]. These species are also known to descend to the ground to feed on humans. The Asian primate reservoir hosts include, cynomolgus monkeys (*Macaca fascicularis*), Southern pig-tailed macaques (*Macaca nemestrina*) and silvered leaf monkeys (*Presbytis cristata*) and possibly green-mitered leaf monkeys (*Presbytis melaphos*) [2,109] (Figure 1). Although only two documented foci of sylvatic DENV transmission have been recognized, if one considers the extent of the geographic range of either vectors and primate reservoir hosts, it is probable that sylvatic DENV transmission occurs, yet undiscovered, in other locations of tropical Africa and Asia [94,110].

To date there is no concrete evidence to document the existence of sylvatic DENV transmission cycle in the Americas. Although several species of New World non-human primates, including *Cebus capucinus*, *Ateles geoffroyi*, *Ateles fusciceps*, *Alouatta palliata*, *Marikina geoffroyi*, *Saimiri orstedii and Aotus trivirgatus*, are susceptible to DENV infection [111], serological surveys (plaque neutralization) of non-human primates in Panama revealed no evidence of enzootic circulation [112]. However, seroconversions among indigenous Ayoreo Indians living in an isolated forested region of Bolivia, where *Ae. aegypti aegypti* are not present, suggests that sylvatic DENV transmission may occur in that region [113]. Recently, reported isolations of all four DENV serotypes in several forest mammals including bats, rodents and marsupials probably represent spillbacks from the human transmission cycle [4,5].

The opportunistic feeding behavior of the arboreal mosquito vectors described above could facilitate transfer of sylvatic DENV from the forest to peridomestic environments (Figure 1). Indeed, in rural areas of Africa and Asia where enzootic vector(s) often reach high densities, DENV is known to transfer between non-human primates and humans. The moist savannahs surrounding sylvan environments in rural areas of Africa and Asia are defined as the ‘zone of emergence’ [114] (Figure 1). In Asia, the studies of Rudnick demonstrated that zoonotic *Ae. niveus* vectors descend to the ground to feed on humans, where *Ae. albopictus* are also abundant, thus allowing the transfer of virus into human habitats [115]. This scenario parallels what has been observed in rural areas adjacent to forests in West Africa, where *Ae. furcifer* is probably the principal bridge vector between forest and human habitats [105].

Currently, almost all human infections are due to DENV strains that circulate exclusively in domestic and peridomestic environments throughout the tropics, where humans serve as the sole amplification and reservoir hosts. In this human cycle, *Ae. aegypti aegypti* mosquitoes transmit DENV [116], while other *Aedes* spp. (for example, *Ae. albopictus*, *Ae. polynesiensis*) serve as secondary vectors [7,99,117,118] (Figure 1). As described above (see Section 4), although *Ae. albopictus* may have been the original vector for human transmission, DENV have fully exploited the highly anthropophilic *Ae. aegypti aegypti* for sustained human transmission. This vector of African origin [119,120] (its ancestral form *Ae. aegypti formosus* utilizes treeholes as larval habitats), exploited the sailing ship trade routes of the 1700s, as well as water storage practices, to infest the tropics almost worldwide [101,121] and in Asia to displace in some locations the native vector [100], *Ae. albopictus*. Furthermore, its domesticated nature, which includes oviposition in artificial water containers leading to large numbers of adult mosquitoes in close proximity to humans, as well as its diurnal feeding pattern and endophilic behavior, permitted it to surpass in epidemiological importance all other *Aedes* spp. Other behaviors of *Ae. aegypti aegypti* that increase its vectorial capacity include its feeble and nervous nature, where feeding interruption at the slightest of movement leads to subsequent return to the same or different host, and its preference for blood over plant nectars for energetic needs. These behaviors increase the potential for this species to transmit a pathogen to several hosts within a very short time [122–124], overcoming the limitation of low oral susceptibility to DENV infection [125] and thus selecting for DENV with higher fitness (viremia) and associated pathogenic potential [27,126].

Transovarial transmission (TOT) has also been suggested as a mechanism of DENV maintenance in both transmission cycles (Figure 1), especially during protracted dry seasons or interepidemic periods. The involvement of TOT in DENV maintenance was demonstrated in nature with the isolation of presumably sylvatic DENV-2 from a pool of *Ae. taylori* in 1980 in Côte d’Ivoire [96], and a year later in Senegal from a pool of *Ae. furcifer* mosquitoes [3]. DENV TOT has also been demonstrated in *Ae. aegypti aegypti* collected from diverse geographic locations and developmental stages [127–136], as well as *Ae. albopictus* [100,137–139], *Ae. mediovittatus* [140], and several members of the *Ae.* (Stegomyia) *scutellaris* group, in which they play an important role in DENV transmission in the Indonesian archipelago and Western Pacific islands (Polynesia) [141].

## 4. Phylogeny as a Tool to Understand DENV Epidemiology and Evolution

The rapid hyperendemicity of the 4 DENV serotypes in the aftermath of World War II was characterized by rapid radiation [68], consistent with a pattern of intense diversification (‘boom and bust’ period) followed by lineage extinction (‘pruning’ period) and clade replacement [72,142–144] (see Section 6 below). This rapid expansion of diversity coincided with explosive human population growth, uncontrolled urbanization, and massive human movement. While early genetic comparisons relied on RNA fingerprinting [145–147] to group DENV strains into topotypes (strains sharing similar spatial distribution), direct viral RNA sequencing allowed for greater efficiency as well as precise characterization of DENV strains. This leap in technology allowed Rico-Hesse to delineate the precise evolutionary relationships of DENV-1 and -2 by introducing the term ‘genotype’ [62], defined as the clustering of DENV viruses with nucleotide sequence divergence not greater than 6% within a given genome region. As technology advanced, automated sequencing allowed for the utilization of complete genes or genomes in obtaining more robust and precise phylogenies, often identifying additional topotypes. Recently a concerted sequencing effort led by The Broad Institute in collaboration with a number of international research centers resulted in an exponential increase the deposition of complete genomic sequences into public databases. Although the availability of complete genomic sequences as well as advanced software tools provide a unique window into understanding how intrinsic factors such as selection pressures, evolutionary rates and population dynamics influence DENV evolution, they often exclude a wealth of previously acquired partial genomic sequences. In this review we will attempt to bridge this reality by constructing a phylogenetic history of all available E gene sequences for each of the four dengue serotypes.

### 4.1. DENV-1

Previous studies based on partial [62,148,149], complete E gene [98,142,144,150–155] or complete genomic sequences [156–160] recognized five distinct DENV-1 genotypes. Our current analysis, based on the complete E gene sequences of 1812 DENV-1 strains, confirms the previously identified lineages. Additionally our expanded sequence dataset allows for a detailed phylogeographic analysis of DENV-1 (Figure 2).

The identified lineages are grouped in 5 genotypes as follows: (a) genotype I, representing strains from throughout Southeast Asia, China and the Middle East (Saudi Arabia). Within this genotype there are several distinct clades associated with outbreaks sharing specific spatiotemporal associations [151,156,157,163]. Of note are the Malaysia and Singapore outbreaks of 2003 through 2008 that resulted in several spillovers into China, Taiwan, Japan and South Korea [154,164,165], as well as a series of Vietnam and China outbreaks sampled between 2001–2003, and 2003–2008 suggesting Cambodia as the major source of DENV-1 outbreaks [157,166,167] (Figure 2). Of interest is the 2004 outbreak in China where a combination of human movement and a natural disaster (typhoon Rananim) led to an explosion of mosquito larval development sites, leading to the outbreak’s rapid spread throughout the region [168]. The reality of DENV outbreak initiations via virus introduction from other localities is best exemplified by the near simultaneous outbreaks of DENV-1 in 2001 in Myanmar and several other distant location throughout the Pacific, which were demonstrated to have been caused by strains from 3 different genotypes [163] (Figure 2). (b) genotype II, representing a couple of strains collected in Thailand during the 1950s and 1960s. These strains have not been sampled since, suggesting that either they have become extinct or are circulating at such a low frequency in niche environments to have escaped surveillance. (c) genotype III (sylvatic?), representing what was thought initially to include the only 2 putative sylvatic isolates collected in Malaysia. The sylvatic nature of these isolates has recently been questioned based on discrepancies in topology by analyses performed on E gene [63] or complete genome sequences [58,94]. The latter analyses do not place the Malaysian canopy DENV-1 isolates in a basal position within this serotype, an observation confirmed by our current analysis of expanded E gene sequences. Therefore, it is possible that these isolates in fact represent spillback events from humans into monkeys rather than true sylvatic isolates. This hypothesis is further supported by the 2005 isolation of human DENV-1 from Malaysia that clusters with the 1972 isolate on the phylogenetic tree [150] (both isolates are depicted in Figure 2 as ‘Malaysia72-05’). Although the authors interpreted this as evidence for the sustained transmission of sylvatic DENV-1 in Malaysia, the fact that both of these strains fall within the mainstream diversity of human DENV-1 suggests that they are ultimately of human origin. (d) genotype IV, representing strains from countries of the Pacific Rim (from Japan, Korea, China, Myanmar, Malaysia and Indonesia) [163], the Western Pacific islands (for example French Polynesia, Nauru, The Philippines and Hawaii), and Australia. Our analysis confirms previous reports that the 2001 DENV-1 outbreak in Hawaii was imported from Tahiti and Samoa [169], and that the 2004 outbreak in China was introduced by a traveler returning from Thailand [168]. Similarly, DENV-1 strains collected during outbreaks from diverse places such as the Caribbean (1977–1985) [170] and the Indian Ocean island of La Reunion (2003–2006), are also grouped within this genotype, underscoring the rapid spread of DENV through frequent human movement and global trade. Lastly, (e) genotype V represents most DENV-1 strains collected in the Americas, strains from West Africa and Asia. The American strains include those collected from Puerto Rico from 1987–1998 [171,172] as well as cases imported into the continental USA at the Mexico/Texas border [173] into the Florida Keys [174] (and reviewed in [175]), Central America (Nicaragua, Mexico, Costa-Rica) [155,176,177], South America (Venezuela, Colombia, Brasil, Paraguay and Argentina) [142,149,160,178–182] and the Caribbean [183]. Several sampled in islands of the Indian Ocean archipelago (Comorros, La Reunion and the Seychelles) during DENV-1 outbreaks in 1993 and 2003–2004 and West Africa during the 1960s during the first documented outbreaks in the region [142,184] are also included.

Overall, the topologies within each DENV-1 genotype are characterized by the basal location of the oldest strains followed by newer isolates, suggesting a pattern of evolution radiating around spatially-defined (geographic) clades.

### 4.2. DENV-2

Although early phylogenetic studies based on partial (prM/E) [62,185] or complete E gene [186] sequences identified 4 major genotypes, subsequent analysis with an expanded E gene dataset revealed the existence of two additional genotypes with restricted geographic (Asia-only) distribution [65,187].

Our current analysis based on a dataset of 1827 complete E gene sequences supports these groupings of six genotypes (Figure 3): (a) Asian genotype I, representing strains from Thailand, Malaysia, Cambodia, Myanmar, Vietnam and Australia. This genotype includes the reference strain 16681, isolated in Thailand in 1964 from a patient with severe dengue disease. Recent studies suggest that the introduction of this genotype into Vietnam from Thailand sometime in the 1990s led to a series of outbreaks and the displacement of the SE Asian/American genotype as the dominant lineage in the region (including Cambodia) [188,189]. This rapid displacement by genotype I was attributed to its fitness advantage (ability to reach higher viremia in humans), leading to an increased rate of human-to-mosquito transmission [189]. (b) Asian genotype II, representing strains from China, Indonesia, The Philippines, Taiwan, Sri Lanka, India, Honduras and Mexico. The Philippine isolates occupy in one clade, which includes two subgroups divided chronologically [187]. The sole Taiwanese isolate represents an imported strain from the Philippines during the 1998 outbreak [190]. This genotype includes the prototype strain New Guinea C (NGC) isolated in 1944 [74]. Several strains isolated independently in different parts of the world (for example, Honduras, Mexico, China) are genetically similar to the NGC prototype strain [65,191–193] (and reviewed in [194]) (Figure 3). Given the rapid DENV nucleotide substitution rates and the limited genetic diversity among these samples, they most likely represent laboratory contaminations. (c) Southeast (SE) Asian/American genotype. This genotype’s topologies suggest a spatial division into two major subclades: (i) all strains collected from Southeast Asia, and (ii) strains collected in Central and South America and the Caribbean over the last 30 years (Figure 3). The founder of this clade was most likely introduced from Vietnam into Cuba in 1981 [195], with profound public health consequences. This Cuban epidemic was associated for the first time in the Western hemisphere with severe disease. Because of the strength of the Cuban public health infrastructure, although there were several thousand cases of severe dengue disease, the case-fatality rate was low [195]. Nonetheless, during the next 30 years strains of this genotype were responsible for major epidemics with increased pathogenicity throughout the Americas. Because of their increased fitness, these viruses displaced less virulent strains of the American genotype from many regions in the Americas [77,196]. (d) Cosmopolitan genotype, previously designated as genotype IV [186], representing strains distributed in a wide geographic area, including East and West Africa, the Middle East, the Indian subcontinent, Indian and Pacific Ocean Islands and Australia. Recently strains of this genotype have been collected in Mexico [153]. As the topologies suggest, there is a spatial subdivision into two subclades (Figure 3): (i) a subclade dominated by strains isolated from the Indian subcontinent [197], Bhutan [198], Sri Lanka, Bangladesh [165] and the Seychelles. The Indian isolates are represented by strains collected after 1971, whereas all pre-1971 Indian strains were genetically similar to the American genotype (see below) [197]. Two clusters of DENV-2 isolated in Saudi Arabia in 1994 are also included, most likely representing two independent introductions into the region by pilgrims from India [199]. A handful of strains from Fiji, Australia, Singapore, Uganda and Malaysia are also included. The latter strains are deeply basal, suggesting a Malaysian origin for this clade. (ii) a subclade that is truly cosmopolitan, characterized by a global distribution with strains sampled as early as 1975 in Indonesia. This subclade is further divided into two distinct groups whose common ancestor appears to have originated in Indonesia (Figure 3). Members of one group appear to have radiated eastward towards East [200] and West Africa [201], but also westward towards the Torres Strait Islands and Australia. Members of the other group appear to circulate widely in the countries of the Pacific Rim, causing outbreaks in Malaysia [202], Singapore [203], the Philippines [187], and Taiwan [165,204,205]. (e) American genotype, previously designated as genotype V [186], representing strains from Central and South America, the Caribbean and older strains collected in the Indian subcontinent and the Pacific Islands. The Indian strains form a distinct lineage within this genotype and include all isolated prior to 1971. This lineage was later replaced by strains of the Cosmopolitan genotype [206]. Isolates of this lineage form 3 distinct subgroups, suggesting that these viruses were introduced at least thrice into India from South America immediately after World War II, a time coinciding with increased movement of ethnic Indians from South America to India and back [197]. Historically, strains of this genotype have been associated with mild DEN, an observation corroborated by experimental data [77,78,81,207,208], and thus are considered to have a low epidemiological impact [192,196,207]. However, recent analyses [209] indicate that outbreaks of this genotype in the 1970s in Puerto Rico [210], Tahiti [211], New Caledonia [212] and Niue [213] included severe dengue disease. Lastly, (f) the sylvatic genotype, representing strains from humans [97,214–219], canopy-dwelling arboreal mosquitoes [3,95,96,105,220] and non-human primates [2,108] collected in West Africa and Southeast Asia as recently as two years ago. This genotype is the most genetically distinct and lies basal to all other DENV-2 lineages, supporting the hypothesis that it represents the ancestral genotype. Within this genotype, the Asian strains are genetically distinct from the West African strains. Recent research has demonstrated that sylvatic DENV evolves in a manner similar to that of human DENV, suggesting that the dynamics of mutation, replication, and selection are broadly equivalent for DENV-2 across its host range [221]. This rapid nucleotide substitution rate is evident in the delineated chronologic divide among all pre-1980 isolates, which form a group distinct from all post-1980 isolates (Figure 3) [97]. In agreement with the historical record, our analysis has confirmed the oscillating nature of the sylvatic amplification cycles in West Africa, in which silent intervals (lack of virus isolates from mosquitoes) of about 8–10 years in length terminate in abrupt spikes of DENV circulation (as detected in 1966, 1974, 1980–1982, 1989–1990, 1999–2000). Human DENV-2 isolations coinciding with these amplification cycles suggests that sylvatic DENV are not confined into the forest but the have the potential to cause limited spill-overs, even in urban settings [97]. These cases revealed that clinical illness resulting from sylvatic DENV infection can be indistinguishable from classic DF resulting from the human transmission cycle. However, two recent cases from Malaysia [215] and Guinea-Bissau demonstrate that sylvatic DENV infection can also cause severe disease [219]. The latter case is striking given that severe dengue disease is rare in Africa, even during urban transmission of typical human strains.

Overall, there is considerable genetic diversity within the DENV-2 genotypes, reflecting their continual divergence and diverse geographic distribution.

### 4.3. DENV-3

In 1994 Lanciotti delineated for the first time 4 distinct DENV-3 lineages corresponding to 4 distinct genotypes [222]: (a) genotype I, representing strains from southeast Asia, the Philippines and the South Pacific islands; (b) genotype II, representing strains from continental Southeast Asia; (c) genotype III, representing strains that spread across Asia, East Africa and into the Americas; and (d) genotype IV, representing strains from Puerto Rico and Tahiti. Several subsequent DENV-3 phylogenies based on the E gene [69,202,223–232], partial gene(s) or gene junctions (e.g., C, prM/E, NS3) [83,233–236] or complete genome [237–239] sequences have confirmed these genotypes.

Our current analysis based on complete E gene sequences of 1208 DENV-3 strains available in GenBank confirms in principle the previously identified genotypes, but also allows for greater resolution to delineate the presence of a new genotype (genotype V) as recently suggested [239–241]. The identified lineages are grouped into 5 genotypes (Figure 4a) as follows: (a) genotype I, representing strains from the maritime areas of Southeast Asia, mainly Indonesia, Singapore, Malaysia, the Philippines and Taiwan [239], and the islands of the South Pacific (e.g., Fiji and Tahiti) [242] (Figure 4a). This genotype includes the majority of isolates from Indonesia from 1973–1983 and 1998–2009 [152,222,243–246], the East Timor outbreaks of 2000–2005 [247–249], two of the earliest samples collected in Malaysia (1974 and 1981) [222,250] and a single isolate from Thailand [143]. The single Thai strain within this genotype raises a few questions, namely was its failure to establish itself: (i) a reflection of regional differences in vector competence? (ii) loss in a competition among genotypes? (the predominant circulating DENV-3 genotype in Thailand, is genotype II). (iii) neutralization due to immunity to closely related strains within a pre-exposed human population, which would severely limit the available pool of susceptible hosts to maintain transmission? (iv) sampling bias? (it is quite possible that strains of this genotype have been circulating as minor variants and thus remained undetected). Overall, it appears that genotype I has been evolving independently in Indonesia [69] over the past 30 years and radiating westward reaching French Polynesia, and northward reaching the Philippines and Taiwan, leading to the first recorded cases of DENV-3-induced severe disease there [239], Tahiti [251] and recently in Brazil [252]. (b) genotype II, representing nearly all strains sampled in Thailand from 1962 until recently in various epidemics (e.g., 1974–1989, 1980–1991, 1988–1991, *etc.*), a single strain from Singapore collected in 1995 (GenBank accession no. AY766104), one isolate from Indonesia sampled in 1998 [243], Taiwan, Vietnam, Bangladesh [253,254], Cambodia, China, Japan and Myanmar (Figure 4a). In contrast to a recent report by Araujo *et al.* [69], where the 1962 Thailand isolate was shown to reside near a common ancestral node of genotype II and III, our analysis shows that this isolate is basal within genotype II. Overall it appears that genotype II has been evolving independently in Thailand over the past 50 years, radiating in countries of the continental Southeast Asia with occasional forays (importations) into Taiwan, China, Bangladesh [69,255] and Norway [256]. (c) genotype III, representing strains from Sri Lanka, India, Japan, Taiwan, Singapore, Samoa, East Africa, Central and South America, the Caribbean and a couple of imported strains to Europe (Figure 4a). This genotype is the most geographically dispersed of all the DENV-3 genotypes. It appears to have emerged somewhere in the Indian subcontinent (in proximity to Sri Lanka), before spreading westward into Africa sometime in the 1980s [257] and leapfrogging into the Americas a decade later. Although in Figure 4a the African strains appear basal to the contemporaneous Sri Lanka isolates, this topology is not highly supported, as the bootstrap value is low. The earliest documented sampling of genotype III in the Americas was in 1994 in Panama and Nicaragua [258,259], but some phylogenetic analyses point to its introduction through Mexico [260] a few years earlier [69]. Whatever the location and time of introduction of this genotype in the Americas might be, it is clear that that these viruses rapidly spread throughout the region by several independent routes of introduction through Central America [236,258,261,262] to the Caribbean [263–267] and to South America [83,223,225,226,268–270] (Figure 4a). The introduction of this genotype into the western hemisphere coincided with explosive epidemics and an increased incidence of severe dengue disease [235,236,260,271]. Strains of this genotype have also radiated eastward as far as Samoa [222] and north as far as Japan, representing importation from Africa [228]. This genotype has also been recently sampled in China, probably imported from the Indian subcontinent [272]. (d) genotype IV, representing strains sampled in Puerto Rico in the early 1960s to late 1970s and in Tahiti. This genotype is the most genetically distinct from all other genotypes of DENV-3 and has only been associated with classic dengue disease [210,273,274]. The position of the sampled strains within this genotype corresponds nicely with the epidemiological record. For example, serologic analysis [275] of strains from the 1963 DENV-3 epidemic in Puerto Rico [273] and the 1965 Tahiti epidemic [274] indicated strong similarities, suggesting that viruses responsible for the Tahiti epidemic were introduced from Puerto Rico (Figure 4a). Furthermore, the low pathogenic potential of these strains may be responsible for a silent epidemic in either location. For example, it is believed that the virus was continually circulating in Tahiti for 4 years until another outbreak of DENV-3 took place in 1969 [276]. Similar to Puerto Rico, the genetic data indicate strains of the 1963 and 1967 Tahitian epidemics (Figure 4a) are closely related, suggesting that the virus was maintained in a silent transmission. Lastly, (e) genotype V, representing the prototype DENV-3 strain (H87–1956) from the Philippines [277], includes an 1973 isolate from Japan, strains from China (sampled in 1987 and 2009) and strains from Brazil sampled in the early 2000s. The lineages within this genotype raise an interesting and intriguing question: why strains sampled in Brazil in 2002–2004 [240,241] and China in 1980 (GenBank accession no. AF317645) and again in 1989 [278] shared very similar sequences with the prototype strain H87 that was isolated nearly 50 years ago in the Philippines [277] (Figure 4b)? Taking into consideration the rapid nucleotide substitution rate that is characteristic of DENV [67,69,71,221,279–282], it is highly likely that these samples represent laboratory contaminations.

Sylvatic DENV-3 are believed to circulate in Southeast Asia based on the seroconversion of sentinel non-human primates [283]. Although no virus isolation has been reported to date, uncorroborated reports suggest that a sylvatic DENV-3 strain may have been isolated in Vietnam in the 1970s [284].

Overall, DENV-3 evolution is characterized by discrete monophyletic and geographically distinct clusters, suggesting spatially contained population bursts with limited co-circulation of various genotypes and occasional but robust gene flow among geographic regions.

### 4.4. DENV-4

While the seminal work of Lanciotti *et al*. [281] and Wang *et al.* [63] identified only 2 genotypes of DENV-4 based on limited E gene sequences, subsequent analyses based on larger datasets delineated 4 major genotypes [58,70,98,280,285]. Our current analysis, based on 418 E gene sequences sampled from 1956 to 2008, confirmed the existence of 4 genotypes and provided greater resolution than the previously identified topologies. These genotypes include: (a) genotype I, representing strains from The Philippines, Thailand, Vietnam, Myanmar, Malaysia, Sri Lanka, India [286,287], and a handful of imported cases in Japan [255], China [256] and Brazil [288,289]. This genotype includes the prototype DENV-4 isolate (H241) isolated in the Philippines in 1956 [277]. (b) genotype II, representing strains from throughout Southeast Asia (Indonesia, Malaysia, Singapore), China, islands of the Western Pacific Ocean, Australia, the Caribbean and the Americas (Figure 5). Recently, strains of this genotype reached Easter Island [290]. As the topologies suggest, there is a spatiotemporal subdivision into two clades: (i) clade I, represents all strains isolated in the Americas and most Asian strains (Indonesia, New Caledonia, Singapore, Malaysia and Tahiti) collected prior to 2000 (Figure 5). The introduction of this genotype from Southeast Asia in the Americas took place sometime in the early 1980s through the Caribbean [291], although Carrington suggested introduction through South America [292]. The former hypothesis is further supported by serologic evidence relating these strains to viruses circulating in French Polynesia a year before the onset of the Caribbean epidemics [147]. From the islands of the Lesser Antilles this lineage spread throughout the Caribbean [293–295] and on to South [296] and Central [153,281] America (and reviewed in [70]); and (ii) clade II, represented by all but three Asian strains collected after 2000 (Figure 5). Within this clade is a distinct lineage of Malaysian strains that AbuBakar suggested may have emerged through intra-serotypic recombination of two ancestral strains [285]. (c) genotype III, represented by five recent Thai strains isolated between 1997 and 2001, appears to be distinct from all other Thai isolates [280]. Lastly, (d) genotype IV, representing the only three known sylvatic DENV-4 strains isolated from sentinel monkeys in Malaysia during the 1970’s. All sylvatic DENV-4 strains are genetically distinct and basal to the clades I–III that represent strains from the human transmission cycle, supporting the notion that they represent the ancestral genotype [63,64,94].

Overall, the strain topologies within each genotype are characterized by the basal location of the oldest strains followed by newer isolates, suggesting a pattern of evolution radiating around temporal rather than spatial (geographic) clades [291,297]. Remarkably, among the two major genotypes (I and II) there appears to be limited genetic exchange; genotype I circulates exclusively in Southeast Asia while genotype II has been evolving independently in Southeast Asia and the Americas for the past three decades. This pattern of isolation has been recently confirmed by other investigators [70].

## 5. Basis of Genetic Diversity of DENV

As stated above, phylogenetic and molecular epidemiology data indicate that DENV consist of four distinct serotypes, namely DENV-1, -2, -3, and -4, which differ at levels similar to those between different flavivirus species [298]. Furthermore, each of the serotypes contains a number of genotypes [62,65,69,70], usually associated with different geographical distributions, and which also contain more detailed genetic infrastructure reflecting the local dispersal of viral strains (shown in Figures 2–5).

The fundamental basis of DENV genetic diversity can be attributed to its error-prone RNA-dependent RNA polymerase, which does not have proof-reading capacity and is thought to produce approximately one mutation per round of genome replication [299,300]. Analyses based on selective pressures, represented as the ratio of nonsynonymous to synonymous substitutions (d*_N_*/d*_S_*), per site suggest that the majority of DENV mutations are deleterious and subject to strong purifying selection (d*_N_*/d*_S_* << 1) [301]. However, genetic variation and population diversity probably allow DENVs to occupy new ecological niches or to adapt to changing niches and selective pressures. Correspondingly, dengue viruses evolve in a rate around or just below 1 × 10^−3^ subs/site/year [67,70,71,197,221,227,302,303], to some extent lower than many acutely infecting RNA viruses that are directly transmitted between vertebrate hosts [279], indicating that DENV may be subjected to stronger evolutionary constraints than the latter.

Another major way of introducing genetic diversity into viral populations is through migration. As discussed above, DENVs have been hyperendemic in Southeast Asia since the 1950s, facilitated by the spread of their mosquito vector, the massive movement of troops and human populations during World War II, as well as uncontrolled urbanization, lack of basic infrastructure and deforestation [7,304]. In the Americas, the cancellation of the vector eradication programs in the 1970s facilitated dengue re-emergence in various regions of Central and South America through the re-introduction and re-colonization of the primary domestic vector *Ae. aegypti aegypti*. As a consequence, between the 1980s and 1990s many Latin American countries evolved from non-endemic (no virus present) or hypo-endemic (one serotype circulation) to hyperendemic [305]. Indeed, growing evidence indicates that DENV gene flow is a common phenomenon and occurs on various scales [188,291,292]. Serotypes and genotypes can be introduced into broad geographical areas where genetically distant strains are already endemic, dramatically increase the genetic diversity and leading to larger fitness variations. In addition to innate viral factors, cross-immunity and antibody-dependent enhancement (ADE) put the fitness of invading strain on a more complicated landscape.

Furthermore, it is noteworthy that the increased host/vector population sizes and densities themselves may help to maintain DENV genetic diversity by increasing the transmission rate and therefore the total viral population size and amount of replication. A large population size not only directly provides the space for larger population diversity, but also reduces the diversity loss due to stochastic events [306].

Finally, although clonal evolution (*i.e.*, the accumulation of mutational changes) has been presumed as the major foundation of DENV evolution, homologous recombination has been suggested as a potentially important factor in increasing genetic diversity in viral population. Indeed, the frequent co-circulation of different serotypes and/or genotypes in one region (see below), together with the fact that *Ae. aegypti aegypti* often engorges multiple times on different hosts [307], makes co-infection of genetically distant DENV strains possible in both mosquito and human hosts. Consequently, concurrent human infection by two or more DENV serotypes appears to be a common occurrence [305] and putative recombinants have been detected within a single mosquito [308]. Several reports have suggested that intra-serotype recombination occurs in all DENV serotypes in different geographical regions [309–316]. Furthermore, the putative evidence of intra-serotypic recombination amongst DENV-4 from independent ancestral lineages (most likely Indonesia 1976 and Malaysia 1969) may have contributed to the emergence of a distinct genotype, representing all Malaysian strains [285]. In contrast, no recombination between serotypes has been detected, suggesting that molecular mechanisms may prevent such event.

However, despite of many reports of putative recombinant DENV strains, no solid evidence has been obtained to attribute the emergence of strains to recombination. In other words, if recombination does occur, it usually confers no significant selective advantage. With the dramatic increase of the amount of sequence data deposited in GenBank through the collaborative effort of the Broad Institute with a variety of international research centers, detection of the frequency of recombination may be evaluated more accurately. However, extreme caution should be exercised when detecting putative recombination events. To date all reported recombination events among flaviviruses have been detected through phylogenetic methods, such as bootscanning. For natural recombination to lead to transmission, three conditions must be met: (a) the recombinant crossover should be demonstrated in a single PCR amplicon following cloning to ensure that it occurs in a single cDNA molecule; (b) the recombination should be demonstrated repeatedly in clonal populations of viable virus (for example, in plaque harvests or limited endpoint dilution isolates); and (c) the recombinant should be stably maintained during post-recombination evolution [98,310].

## 6. Evolution Pattern and its Driving Forces

### 6.1. Dengue Evolution is Characterized by Frequent Lineage Replacement

The evolution of DENV has been associated with its global expansion in the last half of the 20th Century, due to the global spread of the anthropophilic vector *Ae. aegypti aegypti*, the increase of the human population size, uncontrolled urbanization, and expansion of international commerce and travel [317,318]. At local levels, phylogenetic analyses based on longitudinal data have suggested a continuous clade turnover process (although not as striking as in Influenza viruses) whereby individual lineages or entire clades of viruses frequently arise, persist for a period of time, and then disappear [144,319]. Replacement can occur along a single lineage, as is reflected by the ladder-like topology on the phylogenetic tree, due to the regular bottlenecks in viral population size associated with the seasonal fluctuation of vector population size and density [320], purifying selection, which removes all viral strains containing deleterious or slightly deleterious mutations [280,301], or selective sweeps. Often, a clade can disappear after circulating in a particular area for several years and be replaced by a new clade sometimes associated with an epidemic outbreak [163,187]. Related to this clade replacement, co-circulation of different viral strains (including serotype, genotype, or group of viruses within a genotype) is often observed, although with various spatiotemporal associations (see Sections 6.2–6.4 below). Unclear are the detailed mechanisms which lead to clade replacement and which determine the fate of the introduced strains.

### 6.2. The Role of Selection Pressures on Shaping the Evolution and Population Dynamics of DENV

One of the central questions in DENV evolution is how much effect natural selection imposes on its evolutionary dynamics? Theoretically, viral strains with higher virulence (if accompanied with higher and longer in duration viremia leading to greater chance of a mosquito getting infected) and transmission rates from the host, higher infection and dissemination rates in the mosquito vectors, or different antigenicity from previous circulating strains, will be favored by natural selection. Despite the evidence that DENVs are generally under strong purifying selection [301], a few putatively positively selected codons have been detected phylogenetically in several genes (E, NS2A, NS2B, NS4B, and NS5) [65,66,156,197,221,297]. Positively selected codons in the E gene are located either in T- or B-cell epitopes, indicating they are likely involved in escaping the host adaptive immunity, or in regions that affect membrane fusion [64–66]. However, selective pressures acting on different serotypes or genotypes can differ. For example, the Cosmopolitan genotype of DENV-2 exhibits an elevated d*_N_*/d*_S_* value compared to other genotypes, suggesting adaptation during its global spread. Of specific interest is codon E-390, previously identified as a key virulence determinant [321] that could play a role in viral pathogenicity and/or transmissibility due to its location within the distal face of domain III, a region associated with attachment to host cells. Furthermore, the estimation of positively selected codons often varies because it is a function of the spatial and temporal composition of the sample sets [197], suggesting that selection pressure varies in different ecological environments, and often acts on a local scale. For example, a study of DENV-4 evolution over 20 years in Puerto Rico indicated that positive selection (supported by high d*_N_*/d*_S_* values and rapid fixation rates) on NS2A, can at least partially be contributed to lineage turnover [297]. Similarly, positively selected sites on the E gene, which encodes proteins on the viral surface that enable host-cell binding and entry and also provide the primary target for host immune responses, may explain, at least in part, the fixation of the SE Asian/American genotype of DENV-2 in Puerto Rico [322].

Additional experimental lines of evidence have illustrated that fitness variation does exist between different genotypes and even among strains belonging to a single genotype. For example, the Southeast Asian genotype of DENV-2, which was introduced to the Americas in the late 1970s, has effectively replaced the American genotype due to its higher infectivity for *Ae. aegypti aegypti* [77,81,82] and higher viral output in the humans [77,78,207,323]. Similar observations were observed in the Asian 1 lineage of DENV-2, which have entirely displaced the previously dominant Asian lineage in Vietnam, Thailand and Cambodia. A selective sweep is supported by the significantly higher plasma viraemia levels in pediatric patients, which probably leads to more efficient human-to-mosquito transmission [189].

However, although small differences in viral fitness can explain the rapid expansion and fitness of a novel genotype, modeling work suggests that the fate of an introduced strain, even with slight fitness advantage, is ultimately determined by the epidemiological landscape (a combination of factors such as density of mosquito vector population, fluctuation of biting frequency, and a period of cross-immunity, *etc.*) in which it arose [324].

### 6.3. Cross-Immunity as a Driving Force for DENV Evolution

Several phylogenetic studies have explored the traces of positive selection imposed by the cross-immunity by different serotypes on the evolution of DENV*s.* It has been suggested that rapid transmission of DENV populations occurring during concurrent serotype outbreaks may give rise to serologic-escape mutants due to the presence of cross-immunologic pressures [325]. For example, during the 2001 outbreak of DENV-1 in Myanmar, which followed the 1998 outbreak caused by the co-circulation of all four DENV serotypes, six of the eight amino acid substitutions that distinguished [151] the post-1998 DENV-1 strains, as well as all other strains of co-circulating DENV-2, -3, and -4 lineages, were localized in the E protein. This suggested that the presence of strong selective pressures imposed on the circulating DENV-1 strains by cross-reactive antibodies produced against the co-circulating DENV-2, -3, or -4 facilitated their immunologic escape from the strong herd immunity shortly after the 1998 outbreak [151]. The interaction of co-circulating serotypes may also facilitate the intra-serotypic clade replacement. A longitudinal study over a 30-year span in Thailand revealed an inverse correlation between prevalence of DENV-1 and DENV-4 [144]. Increase of prevalence of DENV-1 was often accompanied by an increase in its clade diversity, whereas clade replacements by genotype often occurred with a decline of that serotype, possibly due to their differential susceptibility to cross-reactive immune response ilicited by other serotypes. This observation is further supported by epidemiological models in Thailand [326], suggesting that moderate cross-protective immunity gives rise to persistent out-of-phase oscillations, as shown by DENV-4 circulation during interepidemic years. However, strong or weak cross-protection or cross-enhancement only produces in-phase patterns, as shown by the long-term co-circulation of DENV-1, -2, and -3 [326].

### 6.4. Stochastic Events Play an Important Role in Clade Replacement

The indications of purifying selection (d*_N_*/d*_S_* << 1) in phylogenetic studies along with sporadic occurrences of positive selection as the major selective pressure acting upon DENV genomes [65,301], the co-circulation of different genotypes, rather than immediate replacement after introduction, highlight the importance of environmental and immunological factors than viral factors in determining the occurrence of clade replacement. Possible population bottlenecks between epidemics, as observed during the inter-epidemic periods of 1980 and 1987 in Bangkok, was also proposed as a possible mechanism based on the observation of the low level of fixed amino acid and nucleotide changes in E gene between samples surveyed from the two adjacent epidemics [327]. Similarly, the replacement of the pre-1992 lineage of DENV-3 in Thailand by a local, rather than an introduced strain during the inter-epidemic period was attributed to stochastic events occurring during population bottlenecks [143]. More recently, a comprehensive study examining DENV-1 clade replacement in Myanmar [156], where a long-circulated lineage (genotype III) went extinct following the introduction of two new clades (genotype I) in 1996–1998, suggested that positive selection was not responsible for the extinction of genotype III virus. Rather, it was due to a stochastic event attributable to the low rate of virus transmission during an inter-epidemic period [156].

### 6.5. Antibody-Dependent Enhancement (ADE) and Its Effect on DENV Evolution

Antibody-dependent enhancement (ADE) is a proposed biological mechanism that influences the uptake of DENV by target cells of infection, whereby DENV replication is increased rather than decreased by humoral immunity derived from a previous DENV infection, and has been suggested as one of the mechanisms responsible for severe dengue disease [328,329]. The generation of non-cross-neutralizing antibodies raised against a primary DENV infection leads to the formation of infectious virus-antibody complexes upon a heterotypic DENV infection, which bind to the Fc receptors on the surface of mononuclear phagocytes and facilitate the uptake and replication of viruses [330]. *In vitro* and *in vivo* studies have shown that non-neutralizing antibodies from a previous, heterotypic DENV infection enhance the replication of DENV [331] (enhancement of infection) in rhesus monkeys [329] and humans [26]. In theory, ADE may lead to an increased cell susceptibility (enhanced uptake by macrophages), transmissibility (increased viremia), and mortality (severe dengue disease). The roles of these factors in shaping the evolution and population dynamics of DENV have been discussed in many mathematical modeling studies. Using a dynamic system model of co-circulating dengue serotypes, Cummings *et al.* [332] suggested that ADE could provide a competitive advantage to those serotypes that undergo enhancement compared to those that do not, and that this advantage increases with increasing numbers of co-circulating serotypes. However, the role of ADE may be limited due to the observation that greater levels of enhancement induce large amplitude oscillations in incidence of all dengue virus infections, threatening the persistence of both the enhanced and non-enhanced serotypes [332].

ADE is also thought to be responsible for the large immunological distance between dengue serotypes [64,65,68,333]. Assuming that cross-enhancement acts by increasing the transmission rate of secondary infections, Ferguson *et al.* [76] show that ADE can result in periodic or chaotic coexistence of two pathogen strains. But this model fails to make the connection between ADE and immunological distance, as immunity and enhancement were represented by the same parameter. In an alternative model, Kawaguchi *et al.* [334] assume that increased mortality is the main effect of ADE, and the interplay between ADE and cross-immunity can explain the co-circulating pattern of genetically and immunologically distinct serotypes. However, this model is thought to have used unrealistic parameters, which do not fit the epidemiology and immunology of DENV. To overcome these shortcomings and to clarify the effects of ADE, Adams and Boots [335] incorporated parameters of enhancement in susceptibility, transmissibility and mortality separately and confirmed their results for reasonable levels of susceptibility and transmission enhancement but not for mortality enhancement. The authors also showed that when the two strains have identical basic reproductive rates, no form of enhancement can lead to competitive exclusion. When strains differ in basic reproductive numbers, susceptibility or transmission enhancement allows strains with greater immunological similarity to stably coexist but mortality enhancement forces the strains to be more distinct.

## 7. DENV Evolutionary Rates and Their Constraints

To date, several studies that have examined DENV evolutionary rates, mostly by measuring their substitution rates based on the E gene, have suggested broadly similar rates from 4.6–11.6 × 10^−4^ subs/site/year, for different serotypes, genotypes and transmission cycles [63,67,72,197,221,227,302,303]. These rates are similar to those of many other vector-borne viruses, but in general lower than other acutely infecting RNA viruses that are transmitted directly among vertebrates (usually on the order of ×10^−3^ such as Influenza viruses [279]). These relatively low evolutionary rates, together with the low d*_N_*/d*_S_* values observed on DENV evolution, suggests that DENV may be subjected to certain additional functional or ecological constraints.

The most common idea is that vector-borne viruses are subjected to fitness trade-offs due to their alternating infections between invertebrate and vertebrate hosts. In theory, adaptations to different habitats or hosts are antagonistic; alleles beneficial in one host impair fitness in others. Hence, different selection pressures between vector and primate host may impose additional constraints on vector-borne viruses compared to those transmitted only among vertebrates. Freeing arboviruses from alternating host infections by serial passage *in vitro* [336–342] or *in vivo* [343] consistently leads to an increased fitness in the adaptive cell line or host/vector. Shared mutations in the same position from independent passage series suggested convergent evolution via positive selection during adaptation [98,344]. Confounding results, however, are observed in the fitness change in the bypassed cell line or hosts, as well as in alternate passages. First, despite the fact that most of the studies suggested a fitness loss in the bypassed cell line after serial passage in a single cell line, some studies on vescicular stomatitis virus (VSV) [345] and eastern equine encephalitis virus (EEEV) [346] did not find evidence of fitness trade-offs for the bypassed host. Second, with the exception of Venezuelan equine encephalitis virus (VEEV) [343], all other experimental studies did not observe fitness constraints imposed by alternating passages. In contrast, most of the studies (as mentioned above) suggested that alternating passages increase the viral fitness on both cell lines, in a level similar to the fitness change after serial passages in the specific cell line. The phenotypic change, however, may not be paralleled by genomic changes. It was observed that single host cell passage resulted in the accumulation of more mutations than alternating cell passages without causing obvious fitness deviation [337], suggesting that constraints in evolutionary rates do not necessarily correspond to constraints in fitness. Furthermore, despite the fitness increase, few mutations (if any) were observed based on consensus sequences, suggesting that fitness increases were mediated by minority genomes in the mutant spectrum [343]. To further examine this idea, Coffey *et al.* [342] measured the genetic diversity after serial or alternating passage and its relationship with fitness change and adaptability to novel selective pressures. The results suggested that alternately passaged Chikungunya virus (CHIKV) is associated with greatest fitness increases without drastic changes in genetic diversity, whereas serial passage led to greater increase in genetic diversity, indicating an evolutionary trade-off between maintaining fitness for invertebrate and vertebrate cell cycling. Lastly, these results also provided evidence for a positive correlation between genetic diversity and adaptability to new environment, indicating the role of mutations in minority genomes in the exploration of genetic space and corresponding fitness space in novel selective pressure.

Alternatively, but not mutually exclusively, the observed genetic stability of arboviruses could be attributed to the persistent infection in vectors, where the RNA interference mechanism suppresses viral replication [347]. Experimental studies have provided evidence that mosquito cells place more constraints on the evolution of arboviruses than vertebrate cells. In a study by Chen *et al.* [348], no mutation was observed on the E/NS1 portion of DENV-2 genome after serial passage (20 or 30 times) on C6/36 cells, compared to 4 nucleotide substitutions corresponding to 3 amino acid changes after 30 passages on Vero cells and 1 amino acid change following alternating passages between Vero and C6/36 cells. In a more comprehensive study, Vasilakis *et al.* [336], utilizing both cloned and uncloned DENV, examined whether alternating human-to-mosquito transmission may constrain DENV evolution by imposing constraints on replication in either host, if fitness trade-offs occur. The experimental observations suggested that DENV that were specialized in single host cells (either Huh-7, a human hepatocarcinoma cell line or C6/36, a mosquito cell line), exhibited fitness gains. However, fitness losses were observed in the bypassed cells, and surprisingly viruses passaged in alternating cycles between Huh-7 and C6/36 cells exhibited detectable fitness gains in both cell lines [336]. Moreover, several amino acid changes were observed that were common in both vertebrate and invertebrate cell line passage series suggesting convergent evolution via positive selection. Although these observations support the hypothesis that release of arbovirus from its alternating vector-host transmission cycle will lead to the acquisition of fitness gains for retained vertebrate or invertebrate host, there was limited support to the notion that arboviruses, including DENV, will adopt a fitness compromise. Moreover, a key observation of this study was that DENV specialization in the vertebrate or alternating host cells facilitated the emergence of a qualitatively and quantitatively different mutation spectrum than exclusive passage in the invertebrate host cell line, supporting the notion that positive selection occurs within the vertebrate host [341,342,348].

Lastly, deriving inferences on the impact of host alternation on arbovirus evolution must take under consideration their inherent properties. Specifically, because of the infidelity of their RNA-dependent RNA polymerases (RdRp), arboviruses exist as a swarm of intrahost genetic variants (loosely referred to as ‘quasispecies’). An inherent limitation of most of the studies described so far is the utilization of consensus sequences to monitor the evolution of viral populations, which in reality only reflect the majority nucleotide at any given position of the viral genome and not the spectrum of mutations present. Thus, minority mutant populations that may be characteristic of the RNA virus ensemble can be masked [349]. Utilizing consensus sequences obtained by the amplification and sequencing of RT-PCR amplicons excludes other present mutations (albeit at lower frequencies) from detection that may also play a role in arbovirus evolution. Further support has been demonstrated in recent studies suggesting that the diversity of the intrahost genetic variants can differ in vertebrate hosts and invertebrate vectors [340,350,351], and that these differences can affect viral virulence [352]. Thus, future studies examining the evolution of intrahost genetic variant diversity should capitalize on the continued advances in the efficiency, speed and cost of next generation DNA sequencing that can efficiently sequence simultaneously a large number of RNA viral genomes.

## 8. Conclusions

The human DEN serotypes evolved independently from progenitor sylvatic DENV of each serotype, in a series of parallel divergence events occurring after the establishment of sufficiently large urban populations in the Asia-Oceania region capable to support a human transmission cycle. Consequently, DEN is not new to humanity; it has simply been ushered into our consiousness by the escalation of the global DEN pandemic during the past 60 or so years. This escalation is attributed to human behavior (uncontrolled urbanization, population movement, unsustainable vector control), ecologic (global invasion of the major mosquito vectors) and viral (interaction and evolution of the four serotypes) factors, that have ultimately led to increased viral genetic diversity and disease severity. Understanding the forces that shape DENV evolution and the extent these forces play in the observed shifts to higher virulence in human infections is important in developing effective countermeasures, especially in the absence of a licensed vaccine or antiviral therapies to control the spread of a DENV pandemic.
